# Supplementary material for: Asynchronous magnetic resonance elastography: Shear wave speed reconstruction using noise correlation of incoherent waves
Source: Magn Reson Med. 2022 Oct 27;89(3):990–1001. doi: 10.1002/mrm.29502 (PMC9792433; doi:10.1002/mrm.29502)
Supplement: Supplementary file 1 — DATA S1 MATLAB code used to generate the numerical simulations of Figure 1 [file MRM-89-990-s002.zip › k-Wave/helpfiles/numDim.html]

numDim :: Functions (k-Wave)


# numDim

Return the number of matrix dimensions.

## Syntax

```
dim = numDim(x)
```

## Description

`numDim` returns the number of dimensions of a matrix `x`. Unlike the inbuilt `ndims`, `numDim` returns 1 for 1D vectors, 2 for 2D arrays, etc.

## Inputs

|  |  |
| --- | --- |
| `x` | matrix with unknown number of dimensions |

## Outputs

|  |  |
| --- | --- |
| `dim` | number of dimensions |

## See Also

`ndims`
